# Manganese-functionalized MXene theranostic nanoplatform for MRI-guided synergetic photothermal/chemodynamic therapy of cancer

**DOI:** 10.1515/nanoph-2022-0533

**Published:** 2022-10-10

**Authors:** Dong An, Xin Wu, Yaolin Gong, Wenlu Li, Guidong Dai, Xiaofei Lu, Liangmin Yu, Wen Xiu Ren, Meng Qiu, Jian Shu

**Affiliations:** Frontiers Science Center for Deep Ocean Multispheres and Earth System, and Key Laboratory of Marine Chemistry Theory and Technology, Ministry of Education, Ocean University of China, Qingdao 266100, P. R. China; Department of Radiology, The Affiliated Hospital of Southwest Medical University, Luzhou, 646000, P. R. China

**Keywords:** cancer, chemodynamic therapy, magnetic resonance imaging, MXene, photothermal therapy

## Abstract

Two-dimensional transition metal carbides and nitrides (MXenes) nanosheets with high photothermal conversion efficiency as well as photothermal stability can efficiently generate remarkable hyperthermia for photothermal therapy (PTT) of cancer. However, mono-MXenes cannot exhibit precise diagnosis and treatment to complete ablation of cancer cells in the PTT process. To overcome this dilemma, an “all-in-one” nanoplatform of titanium carbide (Ti_3_C_2_) MXene-based composite nanosheets is developed for magnetic resonance imaging (MRI)-guided multi-modal hyperthermia and chemodynamic tumor ablation, which was achieved by bonding of manganese ion on the surface of Ti_3_C_2_, and then was the functionalized nanosheets was modified by biocompatible PEG (Mn-Ti_3_C_2_@PEG). Due to magnetic and Fenton-like catalytic properties of Mn components, Mn-Ti_3_C_2_@PEG not only acted as the contrast agents for T_1_-weighted MRI (relaxivity value of 1.05 mM^−1^ s^−1^), but also converted cellular H_2_O_2_ into highly toxic hydroxyl radicals (·OH) mediated chemodynamic therapy (CDT). Moreover, Furthermore, Mn-Ti_3_C_2_@PEG can efficiently suppressed tumor-growth by PTT, due to the high photothermal conversion capability and photothermal stability. As a proof-of-concept model, the as-designed Mn-Ti_3_C_2_@PEG nanoplatform shows simultaneous MRI and dual-modal treatment for effective suppression of tumor with minimized side effects both *in vitro* and *in vivo*, indicating the great potential for clinical cancer theranostics.

## 1 Introduction

Precise treatment of tumors to achieve minimal harmfulness to normal tissues has always been a challenging problem in the field of cancer therapy [1–5]. Photothermal therapy (PTT), as an emerging and multidisciplinary strategy for tumor eradication, is developing rapidly and has the potential substitution for traditional clinical cancer treatment. PTT show advantages of minimally invasive, low side effects, high selectivity, good spatiotemporal control, and repeatable treatment, is benefit to maintain the quality of patients’ life and is considered as “tumor green therapy” by the international medical experts. In addition, PTT has synergistic sensitization effect on conventional chemotherapy and radiotherapy leading to significantly enhanced antitumor efficacy. Moreover, PTT can activate the patients’ immunity system and reinforced the body’s own rejective action toward cancer cells [6, 7]. Typically, good PTT system generates hyperthermia using photothermal agents (PTAs) absorb near-infrared (NIR) light well and convert it into heat to elevate temperature exceeded the tumor tolerance temperature to kill cancer cells [8–11]. Layer-structured two-dimensional (2D) transition metal carbides and nitrides (MXenes) as unique PTs have been studied extensively to enhance the therapeutic effects of PTT due to their efficient photothermal conversion capacity and abundant/tunable physiochemical properties [12–17]. In addition, MXene has abundant terminated by –O, and/or –F, which provides the possibility for engineering and functionalizationon its 2D topological surface, endowing them high performance in PTT applications [18–22]. Due to the simple titanium and carbon compositions, though Ti_3_C_2_ alone can photothermal ablation of tumor cells, but cannot exert diagnostic imaging for precise therapy [23–25]. If Ti_3_C_2_ is endowed with imaging guidance function will be beneficial to monitor the ablation process of PTT, so as to realize the integration of diagnosis and treatment for precise therapy of cancer. However, PTT alone cannot achieve complete ablation of cancer cells. As emerging therapy strategies based on nanotechnology, chemodynamic therapy (CDT) have received more and more attention for PTT enhancement to achieve greater combinatorial effect due its many unique merits in increasing tumor specificity and decreasing side effects [26–30].

CDT, as a promising new treatment for cancer, is defined as *in situ* employing the Fenton catalysts in tumor sites to produce high toxic hydroxyl radical (·OH) leading protein, DNA and lipid damage triggered cancer cells death, which could be called “Combat poison with poison”. ·OH is generally produced directly by the *in situ* Fenton or fenton-like decomposition of H_2_O_2_ within tumor microenvironment (TME) mediated by various metal ions (e.g., Fe^2 +^, Cu^+^, Mn^2+^, Cr^4+^and V^2+^) [31–34]. Endogenous CDT is highly selective and does not require the addition of exogenous stimuli in the activation of the reaction. So, CDT has many unique advantages, such as high tumor specificity, low systemic toxicity, and no limitation for the depth of tissue penetration. CDT is sometimes subject to insufficient endogenous H_2_O_2_ in TME and low catalytic efficiency of Fenton or Fenton-like reactions in slightly inappropriate intracellular pH leading tumor ablation is not effective [35]. Thus, combination of PTT and CDT is good way to improve this problem. This “complementary strategy” is practicality and simplicity and beneficial to overcome the deficiency of single therapy and improve the efficiency of collaborative therapy. In addition, temperature is an important factor affecting the rate of chemical reaction, the increased temperature in the tumor area caused by PTT can accelerate the reaction rate of Fenton/Fenton-like response. Therefore, PTT not only directly damages tumor cells, but also promote CDT to enhance synergistic anticancer effect.

Magnetic resonance imaging (MRI) as imaging modalities is featured with individual advantages of higher anatomical structure information on soft tissue at a noninvasive and nonionizing manner, which provides a visualization way to monitor the detail of PTT [36, 37]. Currently, over 50% of clinical MRI examinations require the use of contrast agents (CAs) to improve image quality, which can be divided into T_1_-weighted and T_2_-weighted imaging [38]. T_1_ CAs mainly reduce T_1_ relaxation time and cause an increase in signal intensity (bright contrast), including paramagnetic nanoparticles on metal complexes [39]. Clinical MRI CAs are mainly based on gadolinium-complexes which has potential toxicity inducing kidneys disorders. Hence, it is high interest to develop safe, highly efficient, and cost effective CAs. Manganese (Mn)-based T_1_ contrast agent has the characteristics of high paramagnetism, low toxicity, high biosafety [40]. Mn is a trace element existing in human body and has important physiological and biochemical functions, this is the first non-lanthanide metal reported as MRI CA used for T1 contrast agent enhancement [41–43]. However, most of the Mn ions cannot reach the target position in the organism, resulting in a lot of waste of contrast media. At present, hepatocyte specific mangafodipir trisodium (Mn-DPDP) CA shows good MRI performance in clinical applications, which is the only Mn-based CA approved by FDA [44]. Mn nanoparticles or nanocomposites is one of the effective methods to realize deep penetration and effective *in-situ* enrichment of Mn^2+^ in tumor sites. Rational integration of PTT and Mn^2+^-mediated MRI into one nanoplatform generally can achieve synthetic procedures for further possible clinic translation. Therefore, elaborate design of nanocomposites integrated therapeutic function and imaging function offers tremendous prospects for enhanced PTT.

**Scheme 1:**
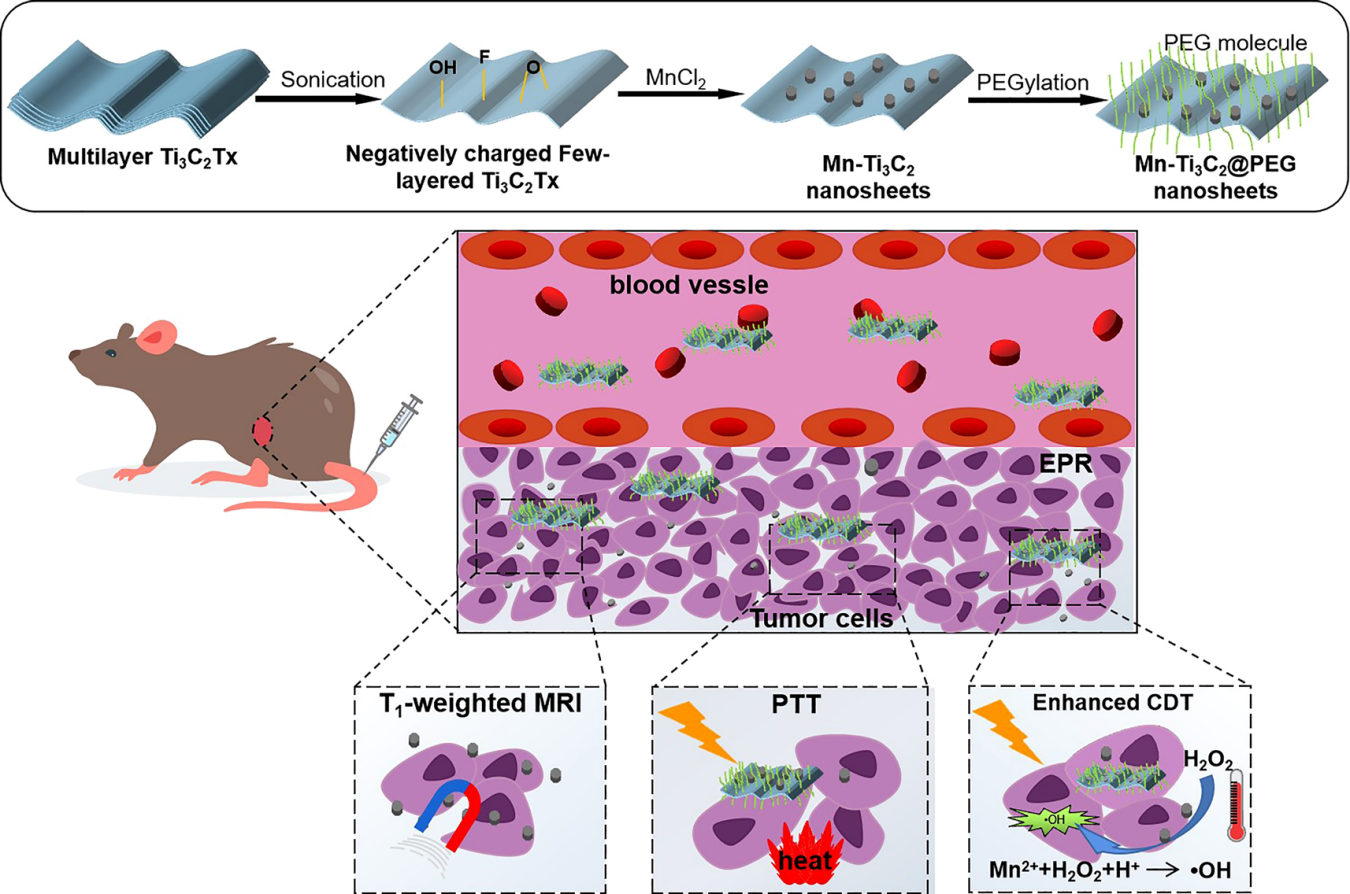
Schematic illustration of the synthetic procedure and MRI-guided efficient photothermal and chemodynamic tumor therapy by Mn-Ti_3_C_2_@PEG.

In this work, we prepared novel 2D Mn-Ti_3_C_2_ MXene composite derived from Mn^2+^ *in situ* bonding with –O and/or –F on the surface of Ti_3_C_2_ MXene, followed by cooperative electrostatic self-assembly with NH_2_-PEG to improve the water solubility and biocompatibility (Scheme 1). This multiple nanoplatform for MRI-guided PTT and CDT of cancer. The photothermal-conversion performance of 2D Ti_3_C_2_ MXene endows as-synthesized Mn-Ti_3_C_2_ to convert NIR irradiation into heat and realize PTT for cancer ablation. Especially, the interaction between Mn-Ti_3_C_2_ and NIR irradiation lead to release of TME-responsive Fenton-like agent Mn^2+^ for H_2_O_2_-supplementing CDT and contrast-enhanced T_1_-weighted MRI-guided therapy. *In vitro* and *in vivo* studies demonstrate that the as-prepared Mn-Ti_3_C_2_@PEG can act as “theranostic nanoagent” to enforce MRI guided synergistic PTT and CDT on combating cancer (Scheme 1).

## 2 Experiments

### 2.1 Synthesis of multilayered Ti_3_C_2_T_x_

Ti_3_AlC_2_ were added into a 40% HF aqueous solution (50 mL), and stirred for 48 h at room temperature. Then, the products were collected by centrifuged and washed for several times with ethanol and water using centrifugation until the pH of the supernatant was above 6. The solution was freeze-dried to obtain multilayered Ti_3_C_2_T_*x*_.

### 2.2 Synthesis of few-layer Ti_3_C_2_T_*x*_

Multilayered Ti_3_C_2_T_*x*_ (500 mg) was immersed to 200 mL of deionized water and sonicated using cell pulverizer (400 W) for 12 h under Ar flow. Then, the dispersion solution was by centrifuged at 5000 rpm for 0.5 h. The dark green supernatant was collected to obtain the few-layer Ti_3_C_2_T_*x*_ colloidal solution, which was freeze-dried to get black powder.

### 2.3 Synthesis of Mn-Ti_3_C_2_

The Mn-Ti_3_C_2_ was prepared by very slowly mixing two dispersion solutions of MnCl_2_ (50 mL, 2 mg/mL) and negative Ti_3_C_2_T_*x*_ nanosheets (50 mL, 2 mg/mL). The mixed solution was sonicated for 10 min and stirred on a magnetic stirrer at room temperature for 4 h. The solution was centrifuged at 6000 rpm for 0.5 h to obtain Mn-Ti_3_C_2_ precipitate, and washed with deionized water and ethyl alcohol three times. The precipitate was dry in a vacuum oven.

### 2.4 Synthesis of Mn-Ti_3_C_2_@PEG

In brief, Mn-Ti_3_C_2_ (50 mg) was be dispersed in 60 mL of deionized water, and then methoxy PEG silane (Mw = 2000, 200 mg) was added. The mixed solution was sonicated for 10 min and stirred on a magnetic stirrer at room temperature for 3 h. The solution was centrifuged for 0.5 h at 9000 rpm to obtain Mn-Ti_3_C_2_@PEG precipitate, removed the supernatant. The precipitate was freeze-dried for standby application.

### 2.5 Photothermal performance of Mn-Ti_3_C_2_@PEG

Photothermal performance of Mn-Ti_3_C_2_@PEG was measured and analyzed by irradiating a EP tube containing 1 mL Mn-Ti_3_C_2_@PEG dispersion with different concentrations. NIR laser was produced using an 808 nm high power multimode pump laser. The temperature and thermal images of the irradiated aqueous dispersion were recorded on an infrared thermal imaging instrument. The extinction coefficient α(λ) and the photothermal conversion efficiency of the Mn-Ti_3_C_2_@PEG is evaluated referred to the previous literature.

### 2.6 Photothermal effect and photothermal cycling stability *in vitro*

1 mL dispersing solution of different concentrations Mn-Ti_3_C_2_@PEG with various concentration (0, 0.0075, 0.015, 0.031, 0.062, 0.125, 0.25, and 0.5 mg/mL) were analyzed by continuously irradiating with 808 nm NIR laser at a power density of 1.5 W cm^−2^ for 5 min, respectively. PBS solution was set as control. An IR thermal imager was used to detecte the photothermal images and temperature variation of all samples. Photothermal conversion cycling-heating of Mn-Ti_3_C_2_@PEG was also carried under 808 nm laser irradiation at 1.0 W cm^−2^ and 1.5 W cm^−2^ five lasers on/off cycles and acquired by IR camera.

### 2.7 Measurement of *in vitro* hydroxyl radical ·OH generation

Methylene blue (MB) is used to evaluate Fenton-like catalytic performance of Mn-Ti_3_C_2_@PEG. Mn-Ti_3_C_2_@PEG at concentration of 0.05 mg/mL was mixed with 13.375 uM MB and 4.895 mM H_2_O_2_. After 30 min, the UV-vis absorption of MB in the mixture was measured by a UV-3600 Shimadzu spectrometer.

The dichloro-dihydro-fluorescein diacetate (DCFH-DA) kit is used to determine intracellular toxic hydroxyl radicals ·OH according to the instructions. 4T1 Cells were planted in six-well plates and incubated with Mn-Ti_3_C_2_@PEG (0–0.5 mg/mL) for 4 h. Then, the cell culture medium was replaced with fresh medium containing the as-prepared Mn-Ti_3_C_2_@PEG. After incubation for 30 min, the cells were treated with DCFH-DA solution and cultured for 0.5 h at 37 °C to detect ROS. The fluorescence intensity of DCFH in cells was observed by fluorescence microscope. For photothermal enhanced group, cells were irradiated by NIR laser for 3 min after co-incubation with as-prepared Mn-Ti_3_C_2_@PEG. Then, the medium was removed and the cells were treated with DCFH-DA solution, which was investigated by fluorescence microscope.

### 2.8 T_1_-MRI performance *in vitro* and *in vivo*

T_1_-MRI properties and T_1_ relaxation time of diluted supernatant were measured by a MR system. 4T1 tumor-bearing mouse were anesthetized by 2% isoflurane in oxygen and placed in prone position. After 0, 10, 20, 30, 40, 50, and 60 min of intravenous injection Mn-Ti_3_C_2_@PEG (40 mg/kg), the Siemens Prisma 3.0 T Signa HDxt superconductor clinical MRI system was used to capture the T_1_ images of tumor area. The parameters were as follows: TR/TE = 2000/3.1 ms, slice thickness = 0.8 mm, 64.3 mm FOV, and matrix size =  144 × 224, and 8° flip angle.

### 2.9 Cytotoxicity assays

Cell counting kit CCK-8 was used to detect the toxicity of Mn-Ti_3_C_2_@PEG to MCF-10A cells and 4T1 cells. Cells were seeded in 96-well plates at a density of 1 × 10^4^/well, and cultured in an incubator for 24 h. After that, the medium containing Mn-Ti_3_C_2_@PEG was added to each well of the experimental group in the form of liquid exchange (the medium containing PBS was added to the control group) and cultured in the incubator. After incubation for 24 h, the medium containing 10% CCK-8 was also added to each well in the form of liquid change. After incubation for 1 h, the absorbance at 450 nm was recorded by microplate reader.

### 2.10 Tumor model

All animal (BALB/c nude mice, 15–22 g, female, 6–8 weeks) procedures comply with institutional animal regulations (the National Institutes of Health guide for the care and use of Laboratory animals (NIH Publications No. 8023, revised 1978)). To obtain tumor-bearing mice, 4T1 cells (2 × 10^6^) were administrated into the right flank of the mice. When the size of the tumor grows about 100 mm^3^, all tumor model of 4T1 tumor-bearing mice were established.

### 2.11 *In vivo* cancer treatment

The subcutaneous tumor model was established by injection of 4T1 cells into the subcutaneous tissue of nude mice. When the tumor reached ∼0.5 cm in diameter, the mice bearing tumor were intravenously injected Mn-Ti_3_C_2_@PEG with the amount of 40 mg/kg of mice weight and irradiated with 808 nm laser for 10 min with the power of 1.5 W/cm^2^. Meanwhile, three control groups were used to assess the therapy efficiency: (a) intravenously injection of 200 μL PBS; (b) intravenously injection of 200 μL PBS and irradiation with 808 nm laser for 10 min with the power of 1.5 W/cm^2^; and (c) intravenously injection of Ti_3_C_2_@PEG with the amount of 40 mg/kg of mice weight and irradiation with 808 nm laser for 10 min with the power of 1.5 W/cm^−2^. The mice weights and tumor sizes were monitored during the treatment period. The tumor volumes were calculated by the equation of Vtumor = (a × b^2^)/2 (a and b represent the maximum and minimum diameter of the tumor). Relative tumor volumes were calculated as *V*/*V*o (*V* and *V*o represent the tumor volume after treatment and initial tumor volume).

## 3 Results and discussion

### 3.1 Synthesis and characterization of Mn-Ti_3_C_2_

Ti_3_C_2_ MXene nanosheets with desirable 2D topology and nanostructures have been extensively and deeply studied because of easy surface engineering and biocompatibility. Particularly, after strict regulation of the composition and structure, 2D Ti_3_C_2_ is more suitable for biomedical applications. Bulk MAX-phase Ti_3_AlC_2_ ceramics were firstly etched in 40% HF solution for 2 days to remove the middle Al layer. Then, the ultrathin Ti_3_C_2_ MXenes nanosheets were fabricateed by ultrasonic treatment of multilayer Ti_3_C_2_ MXenes for 12 h in order to satisfy the biomedical requirements. To integrate Mn^2+^ onto the surface of 2D Ti_3_C_2_ MXene, Mn^2+^ bonded with the interface-exposed –O, –F groups of 2D Ti_3_C_2_ for *in situ* producing Mn-O and Mn-F (Mn-Ti_3_C_2_) through MnCl_2_ introduced into the Ti_3_C_2_ solution. For ensure *in vivo* evaluation and application, polyethylene glycol (PEG) was used to modify Mn-Ti_3_C_2_ nanosheets to enhance biocompatibility and stability of Mn-Ti_3_C_2_ nanosheets. Each component of Mn-Ti_3_C_2_ plays its own role for cancer diagnosis and treatment, Ti_3_C_2_ with high photothermal-conversion capability for photothermal ablation of tumor, and Mn^2+^ for TME-responsive MRI and highly toxic ·OH generation for chemodynamic ablation of tumor.

**Figure 1:**
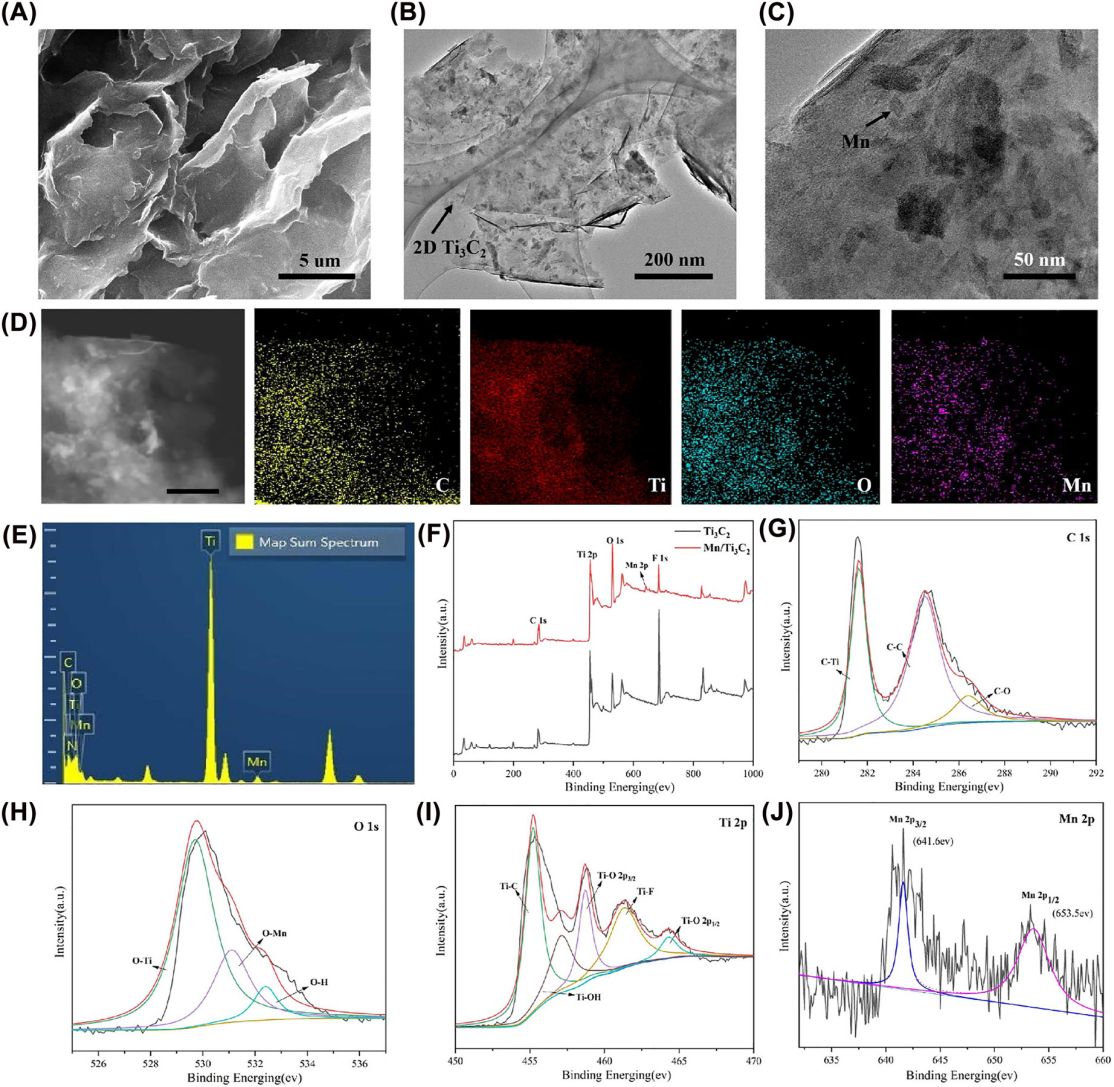
Characterization of few-layer Ti_3_C_2 _nanosheets and Mn-Ti_3_C_2_ composite nanosheets. (A) SEM image of few-layer Ti_3_C_2_ (B) TEM image of Mn-Ti_3_C_2_ (C) TEM image of Mn-Ti_3_C_2_ (D) HRTEM image of Mn-Ti_3_C_2_, and corresponding elemental mapping distribution of C, O, N, and Ti (E) EDS intensity spetra of Mn-Ti_3_C_2_ (F) XPS full spectrums of Ti_3_C_2_, and Mn-Ti_3_C_2_ (G), (H), (I) and (J) are XPS spectras of C1s, O1s, Ti2p and Mn2p of Mn-Ti_3_C_2_, respectively.

**Figure 3:**
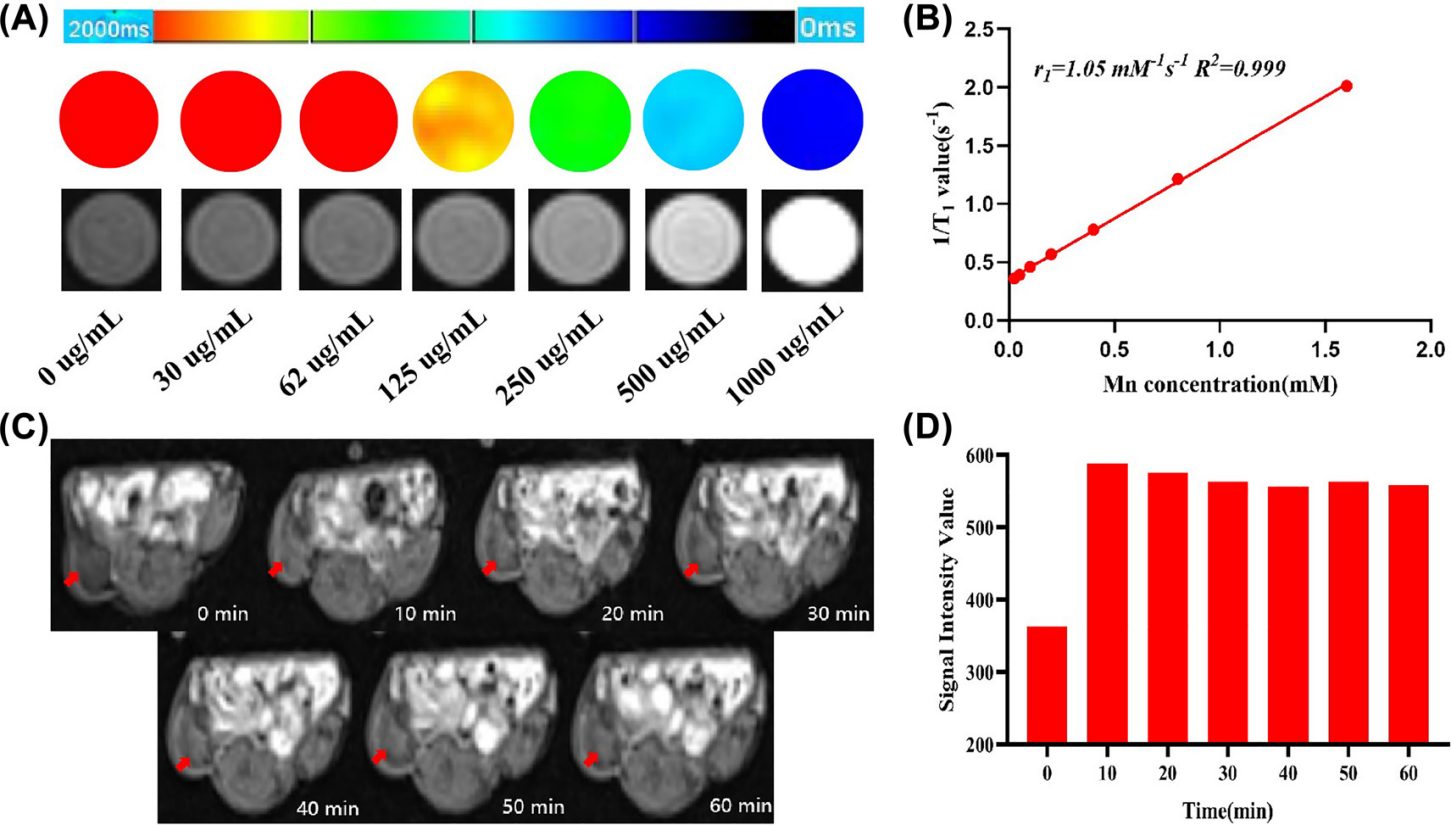
T_1_-weighted MRI of Mn-Ti_3_C_2_@PEG both *in vitro* and *in vivo*. (A) *In vitro* T_1_-weighted MRI and corresponding T_1_ mapping photos of Mn-Ti_3_C_2_@PEG at various concentrations buffer solution after incubation. (B) longitudinal relaxation rate (*r*_1_) as acquired by plotting values of 1/T_1_ over the corresponding Mn-Ti_3_C_2_@PEG concentrations. (C) T_1_-weighted MRI of mouse bearing subcutaneous tumor at 0, 10, 20, 30, 40, 50, and 60 min after intravenous injection of Mn-Ti_3_C_2_@PEG (40 mg/kg per mouse weight), respectively. (D) Quantification of relative tumor contrast collected at different time after administration of Mn-Ti_3_C_2_@PEG. The red arrow on the right back subcutaneously indicates the location of the tumor.

The morphology of the Mn-Ti_3_C_2_ was studied using SEM and TEM. The SEM of the composite presents a well-aligned few-layer microstructure (Figure 1A). As shown in Figure 1B and C, the TEM shows two characteristic morphologies including the large plate-like Ti_3_C_2_ and Mn nanoparticles aggregation. This result indicates that the Mn is intimately reassembled on the Ti_3_C_2_Tx nanosheets. The elemental distribution mappings of Ti, Mn, and O in as-prepared Mn-Ti_3_C_2_ composite nanosheets (Figure 1D) confirmed the coexistence of the Ti and Mn elements and high uniformity of Mn distribution on the surface of Ti_3_C_2_ nanosheets. The signals of Mn is well distributed according to the sites of the Mn on the surface of the Ti_3_C_2_T_*x*_ nanosheets. Besides, the EDS result verifies the elementary composition of the composite containing typical Mn signal, further indicating the Mn functionalized Ti_3_C_2_ (Figure 1E). The surface chemical composition and chemical valence states of the Ti_3_C_2_ and Mn-Ti_3_C_2_ were analyzed by X-ray photoelectron spectroscopy (XPS). C, Ti, O, F, and Mn-atoms are found in the survey spectrum of the Mn-Ti_3_C_2_, while no Mn-atom is found in the Ti_3_C_2_. The amount of surface adsorbed O species in Mn-Ti_3_C_2_ has largely increased but F species were largely decreased after the adsorption of Mn^2+^, that because part of the Mn^2+^ is oxidized by F to form MnO_2_. The C1s peak of Mn-Ti_3_C_2_ can be fitted to three constituent peaks: C–Ti at 281.7 eV, C–C at 284.6 eV, and C–O at 286.4 eV (Figure 1G). The high-resolution spectra of O 1s are shown in Figure 1, where three sub-bands can be found for pure Ti_3_C_2_Tx and Mn-Ti_3_C_2_, the one at 531.1 eV belongs to Mn-O, while the other two peaks at 529.7 and 532.4 eV are for the O species of Ti-O and O–H, respectively (Figure 1H). In Figure 3C, the Ti 2p peak of Mn-Ti_3_C_2_T*x* can be fitted to five constituent peaks: Ti–C at 455.4 eV, Ti–O (2p 3/2) at 459.2 eV, Ti–F at 461.7 eV, and Ti–O (2p_1/2_) at 464.7 eV (Figure 1I). Figure 1J shows Mn 2p in the high-resolution XPS of Mn-Ti_3_C_2_T_*x*_, after grafting the Mn^2+^ onto the Ti_3_C_2_, Mn 2p_3/2,_ and Mn 2p_1/2_ are detected in the Mn-Ti_3_C_2_. Two peaks at 641.6 and 653.5 eV can be assigned to Mn 2p_3/2_ and Mn 2p_1/2_, respectively. The Mn 2p_3/2_ can be deconvoluted into Mn^2+^. The relative contents of Mn^2+^, Mn^3+^, and Mn^4+^ coexist in Mn-Ti_3_C_2_. These XPS results indicate that a large number of Mn is present in the Mn-Ti_3_C_2_.

### 3.2 Photothermal effect

**Figure 2:**
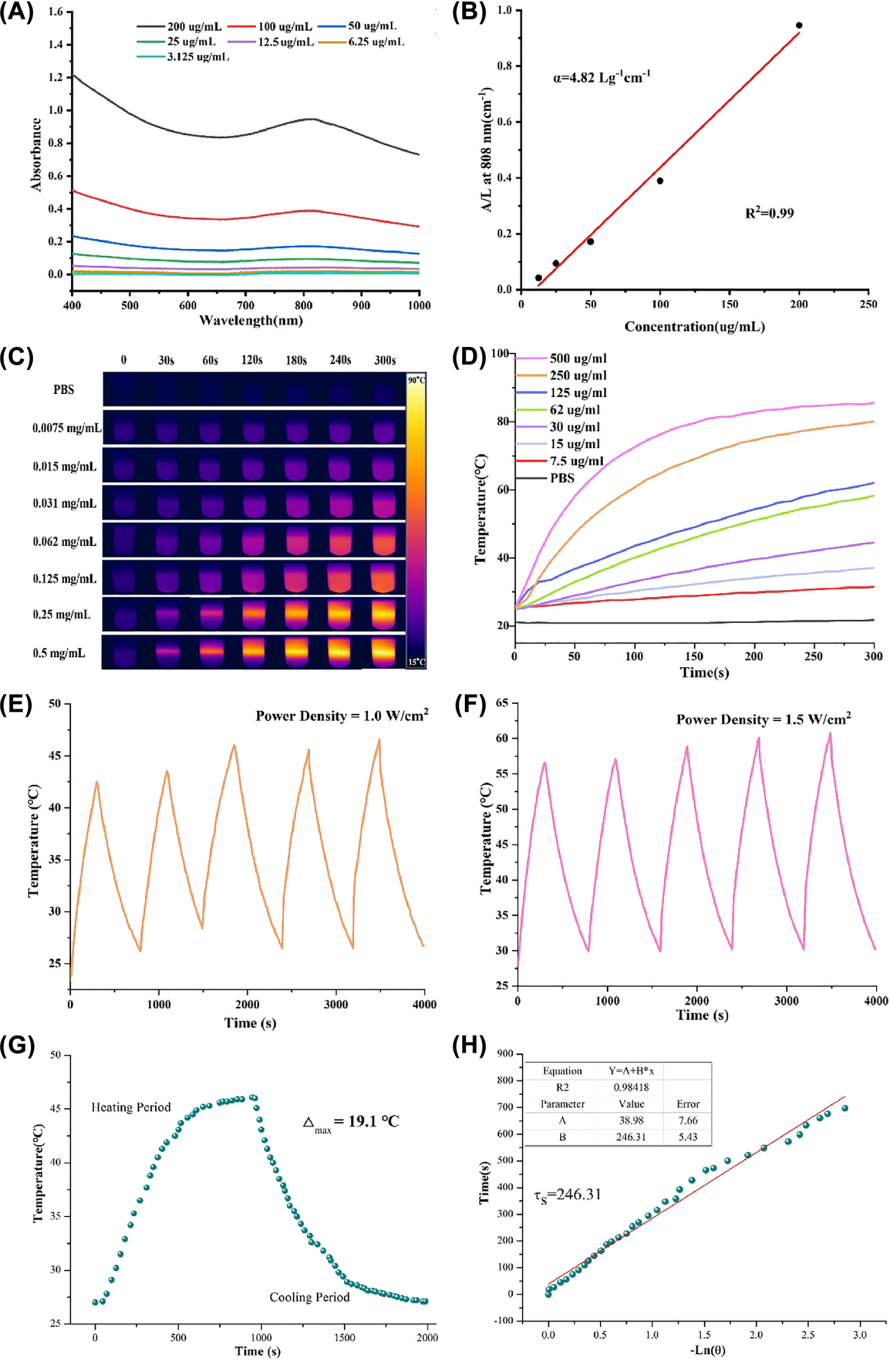
*In vitro* photothermal-property characterization of Mn-Ti_3_C_2_@PEG. (A) UV-vis spectra of Mn-Ti_3_C_2_@PEG in aqueous solution at different concentrations. (B) Absorption coefficient of Mn-Ti_3_C_2_@PEG at 808 nm was recommended to be calculated. (C) The photothermal images of Mn-Ti_3_C_2_@PEG at different concentration under 808 nm laser irradiation (D) change in temperature with time at different concentrations of Mn-Ti_3_C_2_@PEG irradiated with a 808 nm laser at the power of 1.5 W/cm^2^ for 5 min. (E) and (F) recycling-heating profiles of Mn-Ti_3_C_2_@PEG aqueous solution with the concentration of 125 ug/mL after 808 nm laser irradiation at 1.0 W/cm^2^ and 1.5 W/cm^2^ for five laser on/off cycles. (G) Temperature rise and fall curve of Mn-Ti_3_C_2_@PEG under 808 nm laser irradiation (1.0 W/cm^2^) for 2000 s. (H) Fitting curve of cooling time and −Ln*θ* (*τ*_s_ = 246.3).

The photothermal-conversion property of Mn-Ti_3_C_2_@PEG *in vitro* was investigated by measured the temperatures changes of solutions under laser 808 nm laser irradiation. Mn-Ti_3_C_2_ was expected with photothermal-conversion property for further hyperthermia due to the presence of 2D Ti_3_C_2_ with high photothermal-conversion capability in the nanosheets. As shown in Figure 2A, Mn-Ti_3_C_2_@PEG solution displayed concentration-dependent light absorption in the NIR region from UV-vis spectra. The extinction coefficient Mn-Ti_3_C_2_@PEG at 808 nm was tested and calculated to be 4.82 g^−1^ cm^−1^ in Figure 2B, which was higher than that of graphene oxide (3.6 L g^−1^ cm^−1^) and 2D Ti_3_C_2_ alone (3.95 L g^−1^ cm^−1^). *In vitro* photothermal performance of Mn-Ti_3_C_2_@PEG after exposing to an NIR laser (808 nm) at various concentrations under the laser power density of 1.5 W/cm^2^. The infrared thermal images and heating curves show Mn-Ti_3_C_2_@PEG solution have larger temperature increase than PBS after 808nm laser irradiation (Figure 2C and D). The temperature curve shows an obvious upward trend with the increase of Mn-Ti_3_C_2_@PEG solution concentration. After 300 s irradiation, the maximum temperature of Mn-Ti_3_C_2_@PEG aqueous solution with the concentration of 0.5 mg/mL can reach up to nearly 86 °C under the irradiation duration with the power density of 1.5 W/cm^2^, indicating a concentration-dependent photothermal features. In contrast, the temperature of PBS solution only increase 1 °C in after 300 s irradiation at the same conditions, that indirectly confirming Mn^2+^ functionality of Ti_3_C_2_ nanosheets for raising the aqueous temperature. These results prove that Mn-Ti_3_C_2_@PEG can effectively absorb 808 nm laser energy and cause substantial overheating. The photothermal stability of Mn-Ti_3_C_2_@PEG could guarantee the further *in vivo* tumor ablation. In addition, significant temperature changes of Mn-Ti_3_C_2_@PEG under 808 nm laser irradiation in different power density (1.0 and 1.5 W/cm^2^) did not change after 5 cycles of laser on/off for nearly 1 h, suggesting its excellent photothermal stability (Figure 2E). Especially, the photothermal conversion efficiency (*η*) of Mn-Ti_3_C_2_@PEG at 100 μg/mL with a power density of 1.0 W/cm^2^ was evaluated, the calculated results show that the *η* of Mn-Ti_3_C_2_@PEG could reached nearly 41.3%, which is much higher than that of Au nanorods (21%) and is high enough for effective PTT (Figure 2G and H).

### 3.3 *In vitro* · OH* generation ability*

MB served as indicators for verifying production of ·OH in solution to verify the Fenton-like catalytic performance of Mn-Ti_3_C_2_@PEG. When treated with the same concentration (0.2 mM), The absorption value of MB displayed an obvious decrease in Mn-Ti_3_C_2_@PEG and H_2_O_2_ mixed solution, while slight changes in the other solution were observed, demonstrating the good catalytic effect of Mn-Ti_3_C_2_@PEG as Fenton agents (Figure S1). Interestingly, after laser irradiation, in the case of H_2_O_2_ and Mn-Ti_3_C_2_@PEG coexistence, the absorption intensity of MB rapidly reduced within 30 min, the ability for ·OH generation via laser irradiation of Mn-Ti_3_C_2_@PEG solution was still better than that in Mn-Ti_3_C_2_@PEG solution, suggesting that the photothermal effect of Mn-Ti_3_C_2_@PEG displayed promotion effect for ·OH production.

### 3.4 MRI property of Mn-Ti_3_C_2_@PEG

Since Mn^2+^ is a prominent imaging agent for T_1_-weighted MRI, Mn-Ti_3_C_2_@PEG was investigated to be as MRI contrast imaging nanoagent. Mn-Ti_3_C_2_@PEG with concentration of 0–1.0 mg/mL were detected for MRI and relaxation rate measurement. The T_1_-WI and T_1_ mapping imaging for Mn-Ti_3_C_2_@PEG were exhibited in Figure 3A. The T_1_-weighted images become brighter with the increase of Mn-Ti_3_C_2_@PEG concentration, because Mn^2+^ ion due to amount of ion release increased. In addition, as demonstrated in Figure S2, the T_1_-weighted images signal intensity of Mn-Ti_3_C_2_@PEG increased as the concentration increases. As demonstrated in Figure 3B, the longitudinal relaxation ratio (*r*_1_) relaxivity of Mn-Ti_3_C_2_@PEG is linearly fitted and was calculated to be 1.05 mM^−1^ s^−1^.

Encouraged by the great MRI performance of Mn-Ti_3_C_2_@PEG *in vitro*, the mice bearing 4T1 tumors were administrated with Mn-Ti_3_C_2_@PEG as the contrast agents to confirm the accumulation of nanosheets in 4T1 tumor tissues at various time points. Noticeable bright effect of T_1_ weighted images at the tumor site was observed after injection 10–60 min. The tumor regions gradually became brighter after the Mn-Ti_3_C_2_@PEG injection and reached the brightest at 30 min (Figure 3C), suggesting their effective accumulation in tumors. The corresponding T_1_ signal values of the tumor sites were quantified, which rapidly increased after injection of Mn-Ti_3_C_2_@PEG solution, reached the maximum at 30 min (Figure 3D). At the 10-60 min post injection, the T_1_ signal value of the tumor region was almost the same, which might indicate the T_1_ weight signals can continuous close to an hour or more in the tumor region of Mn-Ti_3_C_2_@PEG. These results clearly verified Mn-Ti_3_C_2_@PEG were feasible MRI contrast agents in living systems.

### 3.5 Intracellular generation ·OH and biotoxicity

**Figure 4:**
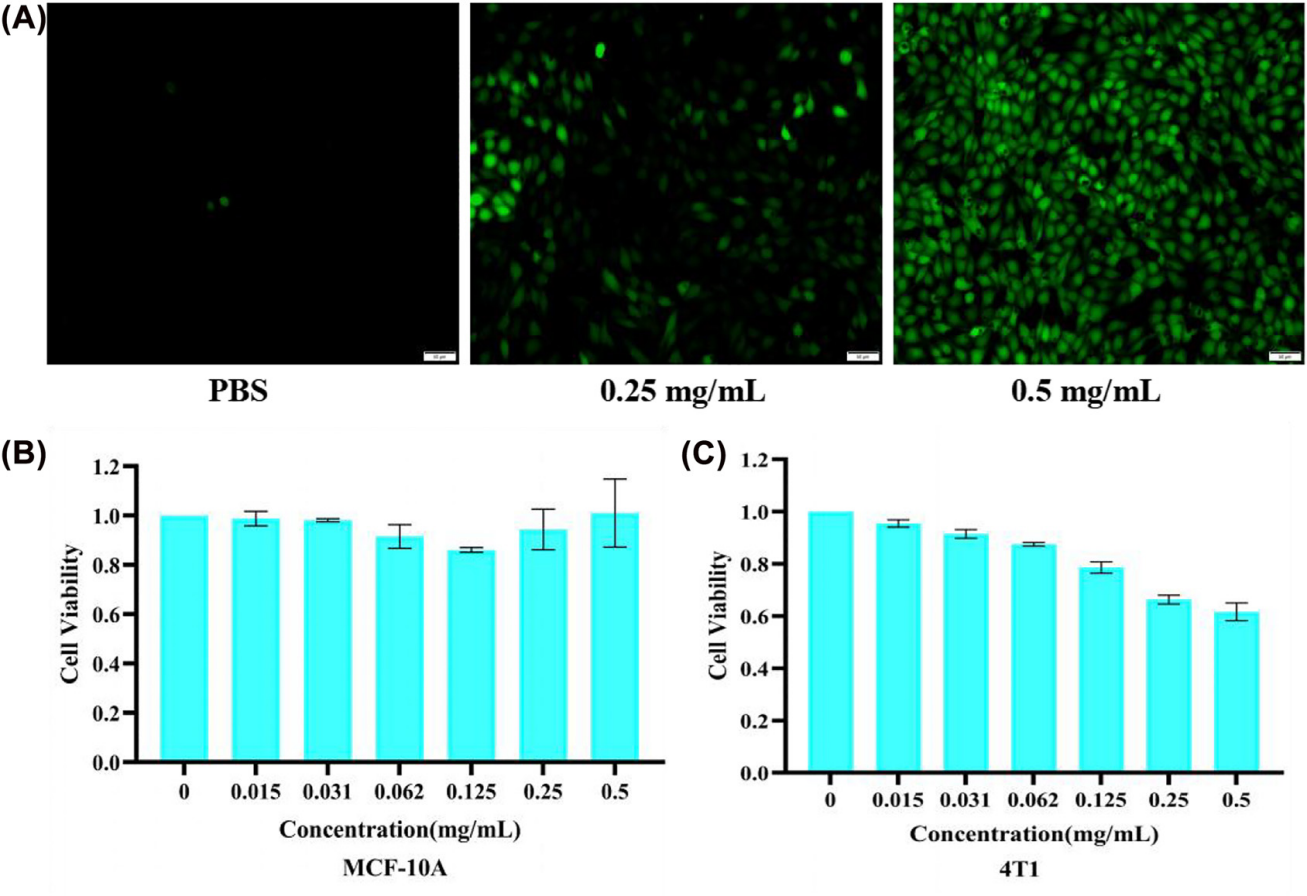
Intracellular generation ·OH and biotoxicity of Mn-Ti_3_C_2_@PEG. (A) Fluorescence microscope images of 4T1 cells incubated with ROS fluorescence probe DCFH-DA and different amount of Mn-Ti_3_C_2_@PEG. Scale bar is 50 μm. Relative viabilities of (B) MCF-10A and (C) 4T1 cells after the co-incubation with Mn-Ti_3_C_2_@PEG at different concentrations for 24 h.

To assess the CDT therapeutic efficacy mediated by the fenton-like reaction *in vitro*, 4T1 cells were incubated with Mn-Ti_3_C_2_@PEG in the presence or absence of H_2_O_2_ (100 μM). DCFH-DA served as indicators for verifying production of intracellular ·OH to verify the Fenton-like catalytic performance of Mn-Ti_3_C_2_@PEG. Detecting intracellular ·OH levels by using DCFH-DA (high green fluorescence means high ROS levels). The PBS group showed no obvious fluorescence. However, after the addition of Mn-Ti_3_C_2_@PEG, a clear green fluorescence appeared, which indicating abundant ·OH were generated. The higher the Mn-Ti_3_C_2_@PEG concentration, the more ·OH were produced (Figure 4A). Thus, Mn-Ti_3_C_2_@PEG showed excellent catalysis ability of Mn-Ti_3_C_2_@PEG to generate ·OH.

It is important to investigate the biocytoxicity, biocompatibility, and biosafety of Mn-Ti_3_C_2_@PEG in cell for their potential clinical research and applications. To evaluate the biosecurity of Mn-Ti_3_C_2_@PEG, we explored the cytoxicity of Mn-Ti_3_C_2_@PEG to normal cells. MCF-10A was chosen as normal cells. After coincubation of Mn-Ti_3_C_2_@PEG with MCF-10A and 4T1 cells at elevated concentrations for 24 h. As shown in Figure 4B, there are no obvious inhibition effects on the MCF-10A cell viability even at a high evaluated concentration of 0.5 mg/mL, indicating its excellent cyto-compatibility to normal cells. However, the as-fabricated Mn-Ti_3_C_2_@PEG exhibited stepped cytotoxicity to 4T1 Cells as concentrations increased. The cell viability of 4T1 diminished to 61% after treated with Mn-Ti_3_C_2_@PEG (0.5 mg/mL), which exhibited stronger cytotoxicity to 4T1 cells owing to chemodynamic reaction occurred in cancer cells.

### 3.6 *In vivo* synergetic PTT and CDT effect

**Figure 5:**
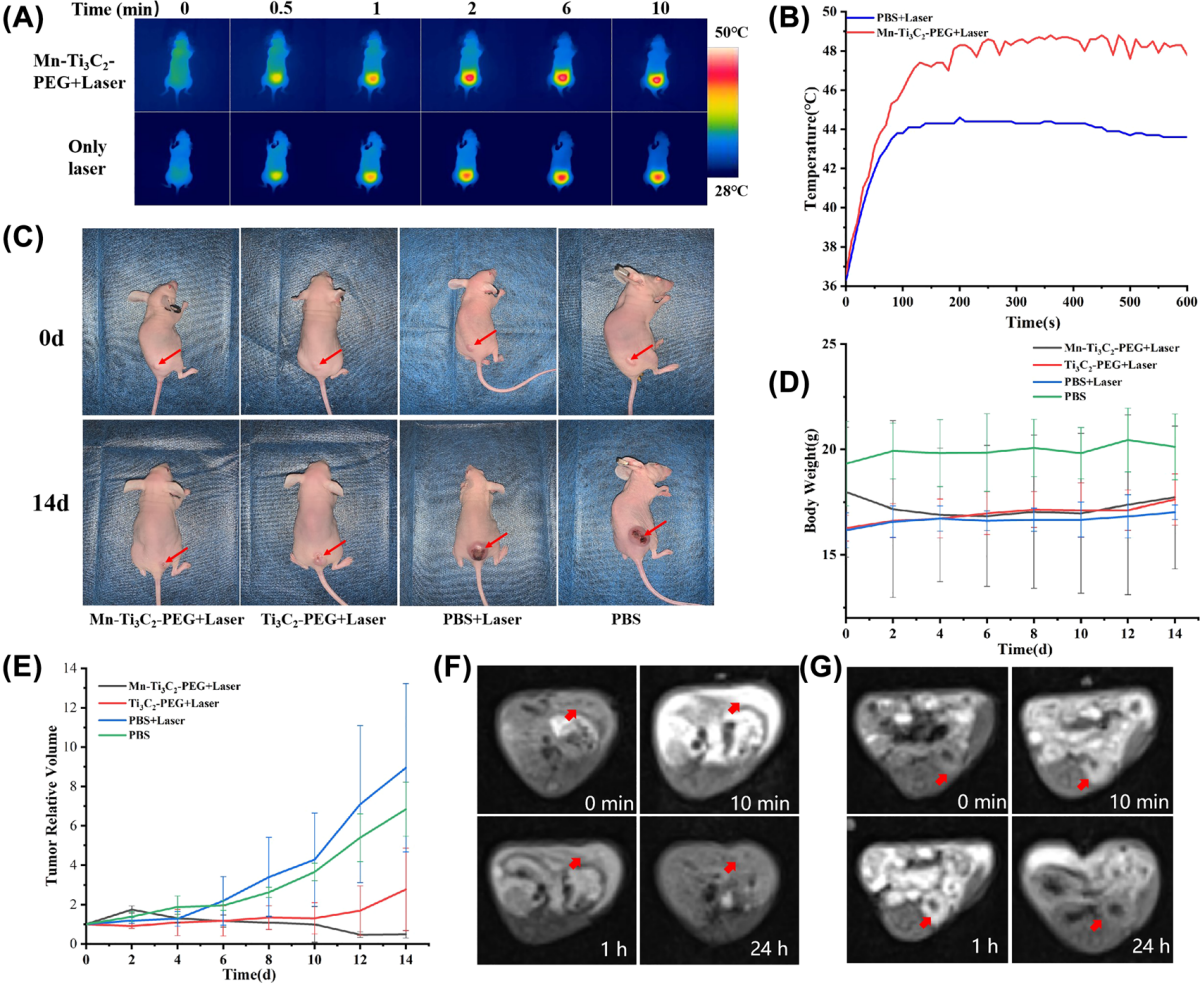
The infrared thermal pictures (A) and the corresponding temperature changes (B) at the tumor site after intravenously injected with Mn-Ti_3_C_2_@PEG under NIR-II laser illumination (808 nm) at different time. (C) Digital images of tumors in mice from each group at the end of various treatments after 14 d. (D) Body weight time-dependent body-weight curves of 4T1 tumor-bearing nude mice of four groups after receiving different treatments, including PBS, NIR laser only, Ti_3_C_2_@PEG + NIR laser and Mn-Ti_3_C_2_@PEG + NIR laser. (E) Time-dependent tumor-growth curves of four groups (PBS, NIR laser only, Ti_3_C_2_@PEG + NIR laser and Mn-Ti_3_C_2_@PEG + NIR laser) after receiving different disposes (F) T_1_-weighted MRI of the liver area in 4T1 tumor-bearing nude mouse at 0, 10 min, 1 h and 24 h after intravenous injection of Mn-Ti_3_C_2_@PEG, respectively. The red arrow indicates the location of the left hepatic lobe. (G) T_1_-weighted MRI of the left kidney in 4T1 tumor-bearing nude mouse at 0, 10 min, 1 h and 24 h after intravenous injection of Mn-Ti_3_C_2_@PEG, respectively. The red arrow indicates the location of the left kidney.

Mice bearing 4T1 tumors were carried out to conduct antitumor efficacy on tumor inhibition of Mn-Ti_3_C_2_@PEG (20 mg/kg)-mediated CDT/PTT synergistic therapy. Mice bearing 4T1 tumors were administrated with Mn-Ti_3_C_2_@PEG by tail intravenous injection and irradiated by NIR light. The 4T1 tumor bearing mice were randomly injected three groups (PBS, Ti_3_C_2_@PEG and Mn-Ti_3_C_2_@PEG). After intravenous administration of different group for 12 h, the mice were treated with an 808 nm laser (1.5 W/cm^2^, 10 min) at the tumor region. After 14 days, the changes of body weight and tumor volume were monitored and counted without extra injection and laser irradiation. The temperature was recorded to confirm the NIR PTT effect during laser irradiation. Comparing to that of the PBS, the local temperature of tumor in Mn-Ti_3_C_2_@PEG-injected mice was raised significantly from 36 °C to 48 °C treated with Mn-Ti_3_C_2_@PEG under 808 nm laser irradiation for 10 min, which was sufficiently high for tumor ablation. However, the maximum temperature of NIR laser only group was just about 43.6 °C under the same laser power density, indicating that the good photothermal conversion performance *in vivo* of the as-prepared Mn-Ti_3_C_2_@PEG for PTT (Figure 5A and B). The mice weights were recorded every 2 days after the different treatments, which showed weight changes among these four groups. During the treatment period, the body weights of all mice nearly maintained at the same level, indicating the minimal side effect of Mn-Ti_3_C_2_@PEG (Figure 5C and D). The Mn-Ti_3_C_2_@PEG group was more efficient in tumor suppression compared to the Ti_3_C_2_@PEG groups due to the CDT effects. The strongest anti-tumor efficacy was achieved in the Mn-Ti_3_C_2_@PEG + NIR group due to synergistic effect of PTT and CDT, which supported the feasibility of Mn-Ti_3_C_2_@PEG as a highly efficient cancer therapeutic agent. The length and width of the tumors were measured by a digital caliper every 2 days during the next 2 weeks to form tumor growth curves (Figure 5E). Mice model treated with PBS and laser displayed rapid tumor growth. Notably, the tumor growth rate of Mn-Ti_3_C_2_@PEG + Laser group was almost inhibited, revealing the high synergistic CDT/PTT therapy efficiency. We further utilized Mn-Ti_3_C_2_@PEG to assist liver and kidney MRI to evaluate its contrast capacity *in vivo*. T_1_-weighted contrast images were acquired before and after intravenous injection of Mn-Ti_3_C_2_@PEG with the dosage of 40 mg/kg body weight of mouse on a T_1_ MRI scanner. After intravenous injection, we indeed observed signal increase in liver (Figure 5F) and kidney region and (Figure 5G). Signal intensity is calculated by choosing liver and kidney as the region of interests (ROIs) to quantify the contrast enhancement. There results suggest Mn-Ti_3_C_2_@PEG could be as a candidate to assist to obtain comprehensive information in T_1_-weighted MRI and achieve tumor diagnosis.

## 4 Conclusions

In summary, manganese-functionalized 2D Ti_3_C_2_ nanosystem have successfully established for T_1_-weighted MRI-guided synergetic PTT and CDT of tumor. Mn-Ti_3_C_2_@PEG was activated for *in situ* surface engineering bond of Mn^2+^ on the surface of 2D Ti_3_C_2_ nanosheets. One hand, Mn-Ti_3_C_2_@PEG acted as the high-performance contrast agents for simultaneous T_1_-weighted MRI and ·OH mediated Fenton-like reaction to exert CDT due to the existence of Mn^2+^ composition. On the other hand, the constructive photothermal reagent Ti_3_C_2_ nanosheets promoted Mn-Ti_3_C_2_@PEG to generate excellent photothermal-conversion performance under 808 nm laser irradiation, which has realized significant ablation of tumor cells by generated local heat. This as-designed composite realized MRI-guided synergistic PTT and CDT to completely inhibition of tumors growth *in vivo* via systematic evaluation. Therefore, the Mn-Ti_3_C_2_-based all-in-one nanoplatform with simultaneous MRI guided synergistic PTT/CDT treatment might be a highly promising nanoagent for clinical cancer theranostics.

## Supplementary Material

Supplementary Material Details
