# Supplementary material for: Manganese-functionalized MXene theranostic nanoplatform for MRI-guided synergetic photothermal/chemodynamic therapy of cancer
Source: Nanophotonics. 2022 Oct 10;11(22):5177–88. doi: 10.1515/nanoph-2022-0533 (PMC11501808; doi:10.1515/nanoph-2022-0533)
Supplement: Supplementary file 1 — Supplementary Material Details [file j_nanoph-2022-0533_suppl_001.docx]

**Supporting information**

**Manganese-Functionalized MXene Theranostic Nanoplatform for MRI-guided Synergetic Photothermal/Chemodynamic Therapy of Cancer**

Dong An^a,#^, Xin Wu^b,#^, Yaolin Gong^b^, Wenlu Li^b^, Guidong Dai^b^, Xiaofei Lu^b^, Liangmin Yu^a,^*, Wen Xiu Ren^b,^*, Meng Qiu^a,^*, Jian Shu^b,^*

^a^ Frontiers Science Center for Deep Ocean Multispheres and Earth System, and Key Laboratory of Marine Chemistry Theory and Technology, Ministry of Education, Ocean University of China, Qingdao 266100, P. R. China.

^b^ Department of Radiology, The Affiliated Hospital of Southwest Medical University, Luzhou, 646000, P. R. China.

## 1 Materials

## X-ray photoelectron spectroscopy (XPS) spectrum was recorded by ESCAlab250 (Thermal Scientific). UV-vis-NIR absorption spectra were recorded by UV-3600 Shimadzu UV-vis-NIR spectrometer with QS-grade quartz cuvettes at room temperature. The optical absorbance per cell length (A/L) was measured from the optical absorbance intensity at 808 nm. The Ti3C2 extinction coefficient was extracted from the slope of a plot of A/L versus concentration from Beer’s law (A/L = αC). Scanning electron microscopy (SEM) images and corresponding element mapping were obtained on a field-emission Magellan 400 microscope (FEI Company). Transmission electron microscopy (TEM) images and corresponding EDS spectrum were obtained on a JEM-2100F electron microscope operated at 200 kV. The Ti_3_C_2_ concentration was measured by inductively coupled plasma atomic emission spectroscopy (ICP-AES, Agilent Technologies, US). The confocal laser scanning microscopy (CLSM) images were obtained in FV1000 (Olympus Company, Japan).

2


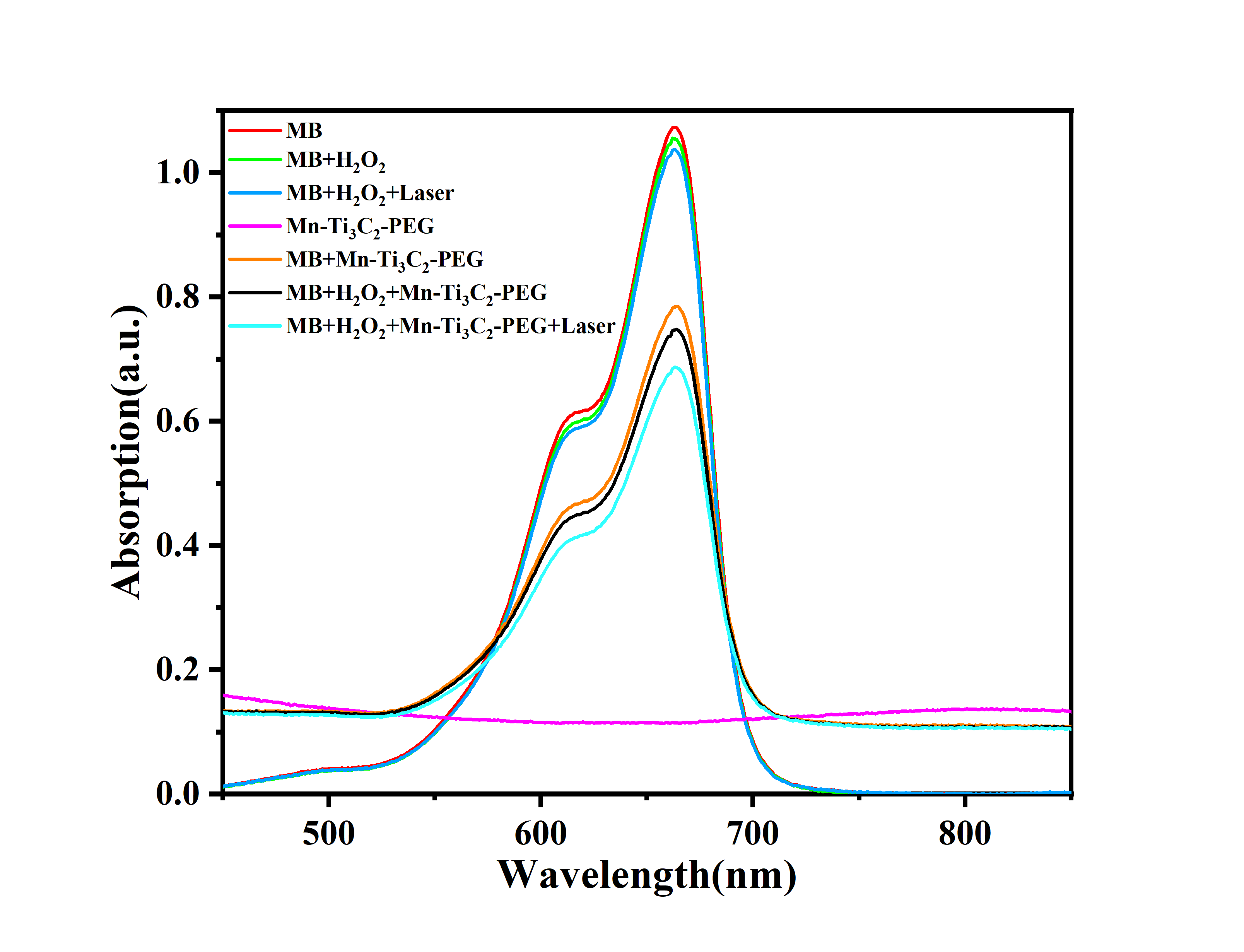


**Figure S1**. 1 UV-vis absorption of different experimental groups to test the Fenton reaction of Mn-Ti_3_C2-PEG


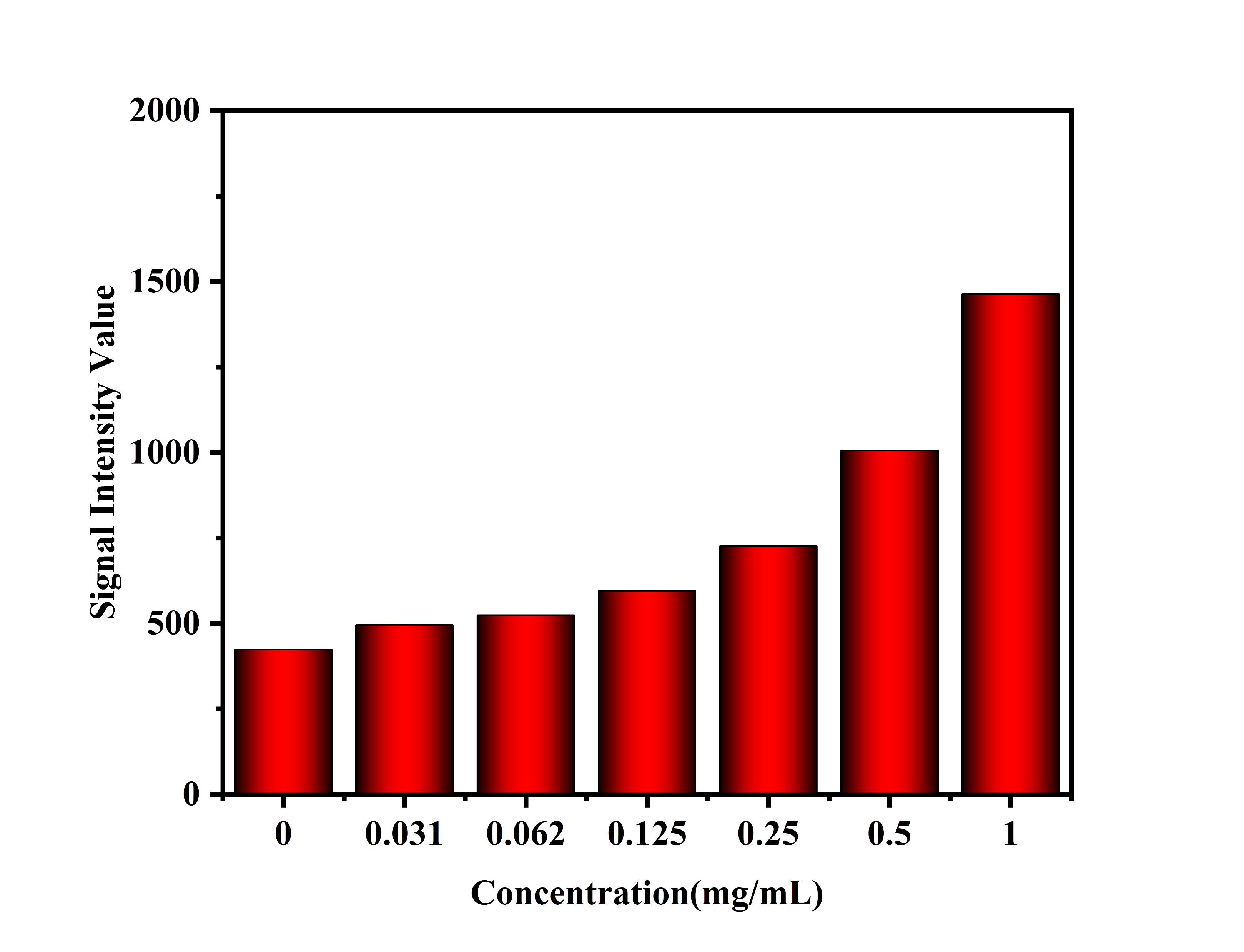


**Figure S2**. T1WI signal intensity of different concentration of Mn-Ti3C2-PEG.


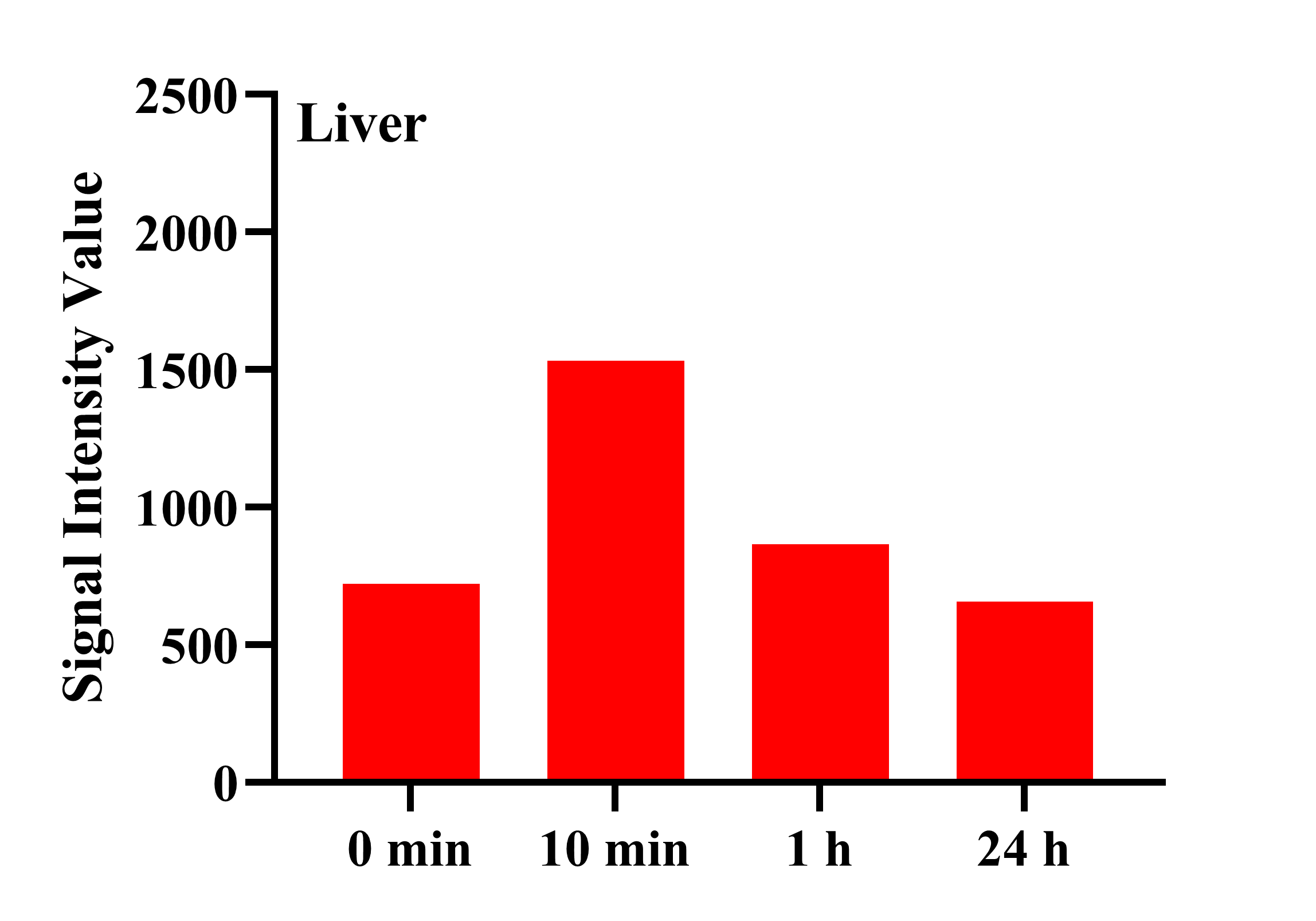


**Figure S3**. T1-weighted MR images signal intensity of the liver area in 4T1 tumor-bearing nude mouse at 0, 10 min, 1 h and 24 h after intravenous injection of Mn-Ti3C2-PEG , respectively. the red arrow indicates the location of the left hepatic lobe.


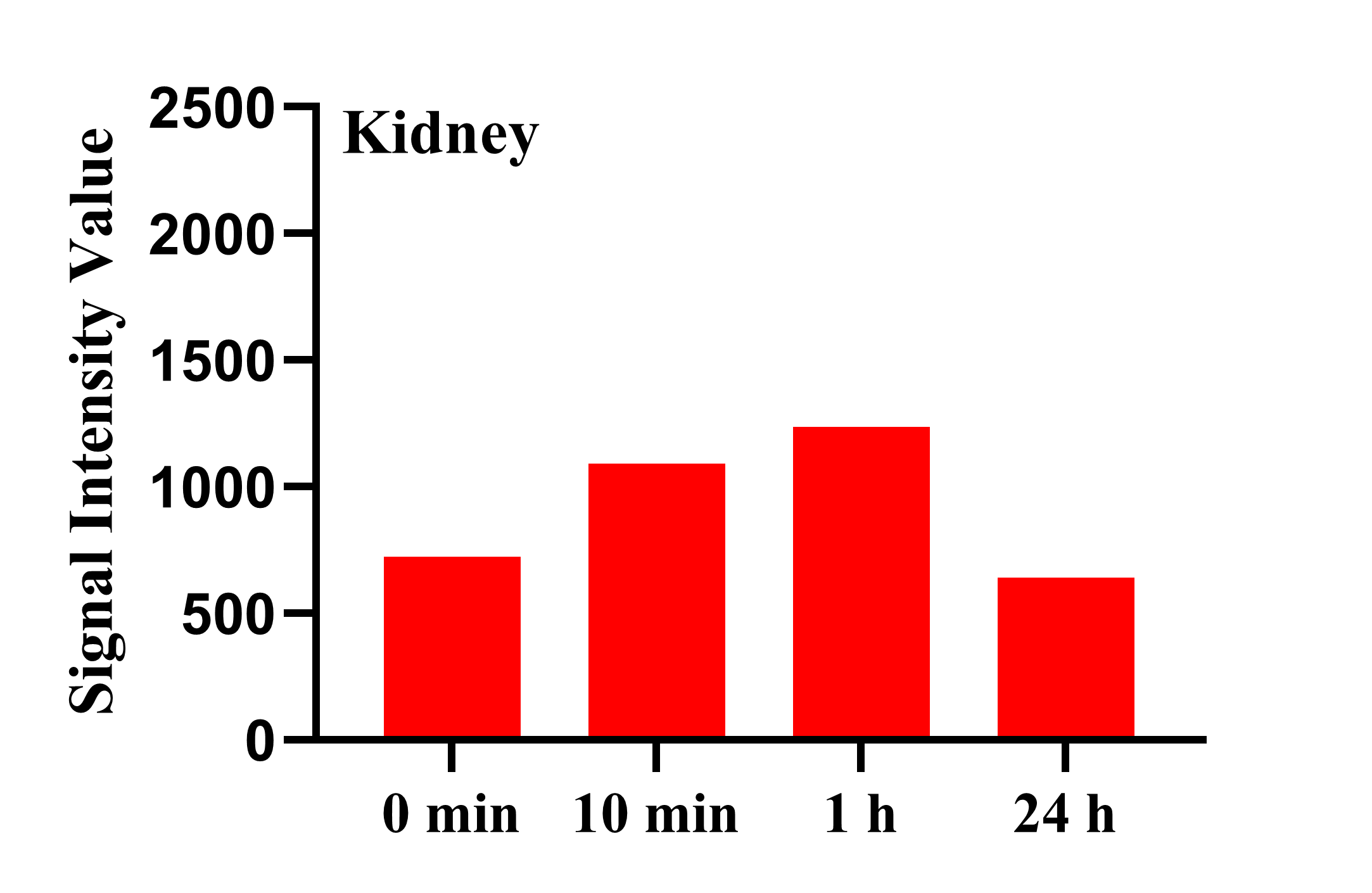


**Figure S4**. T1-weighted MR images signal intensity (b) of the left kidney in 4T1 tumor-bearing nude mouse at 0, 10 min, 1 h and 24 h after intravenous injection of Mn-Ti3C2-PEG , respectively. the red arrow indicates the location of the left kidney.
